# Supplementary material for: Distinct Adipogenic and Fibrogenic Differentiation Capacities of Mesenchymal Stromal Cells from Pancreas and White Adipose Tissue
Source: Int J Mol Sci. 2022 Feb 14;23(4):2108. doi: 10.3390/ijms23042108 (PMC8876166; doi:10.3390/ijms23042108)
Supplement: Supplementary file 1 [file ijms-23-02108-s001.zip › Supplementary_Figure S1.pdf]

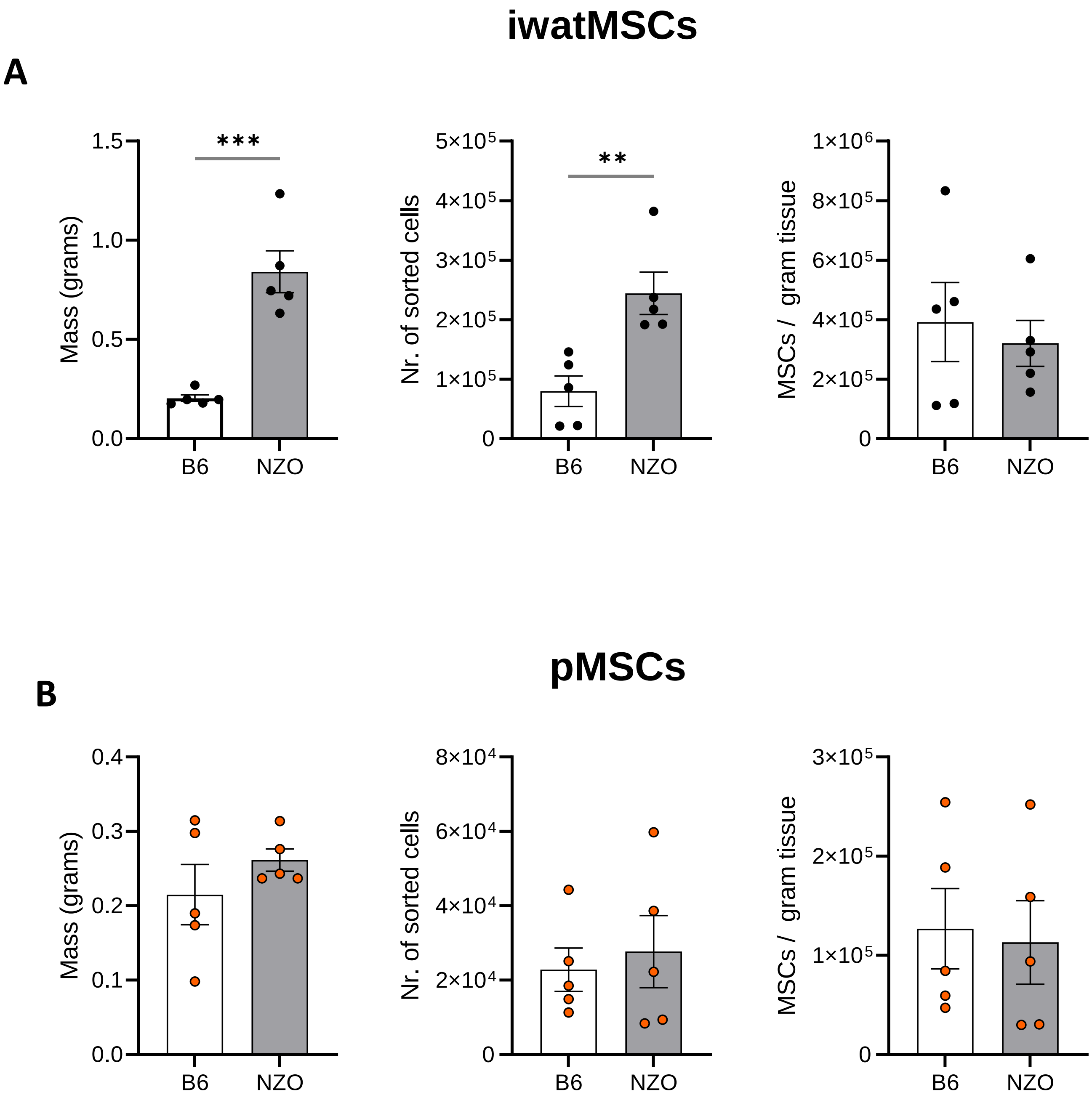

Supplementary Figure S1. pMSCs and iwatMSCs from B6 and NZO mice do not differ in their relative cell count. (A) Comparison of B6 and NZO pancreas weight, sorted MSCs and relative MSC count per gram tissue. **(B)** Comparison of B6 and NZO iWAT weight, sorted MSCs and relative MSC count per gram tissue (n=5 per group). Data shown as mean ± SEM. Statistical difference was calculated using Student's *t*-test with Welch's correction ($p<0.05$, ***$p<0.001$).
